# Left ventricular deformation associated with cardiomyocyte Ca^2+^ transients delay in early stage of low-dose of STZ and high-fat diet induced type 2 diabetic rats

**DOI:** 10.1186/s12872-016-0220-8

**Published:** 2016-02-16

**Authors:** Xiao-Ying Liu, Fu-Cheng Liu, Chun-Yu Deng, Meng-Zhen Zhang, Min Yang, Ding-Zhang Xiao, Qiu-Xiong Lin, Shi-Ting Cai, Su-Juan Kuang, Jing Chen, Shao-Xian Chen, Jie-Ning Zhu, Hui Yang, Fang Rao, Yong-Heng Fu, Xi-Yong Yu

**Affiliations:** Guangdong Cardiovascular Institute and Medical Research Center, Guangdong General Hospital, Guangdong Academy of Medical Sciences, 106 Zhongshan Er Road, Guangzhou, Guangdong 510080 P.R. China; Department of Cardiology of the First Affiliated Hospital, Jinan University, Guangzhou, 510630 P.R. China; Institute of Molecular and Clinical Pharmacology, Guangzhou Medical University, Guangzhou, 511436 P.R. China

**Keywords:** Diabetic cardiomyopathy, Diabetes mellitus, Speckle tracking echocardiography, Rat, Ca(2+) transient, CaMKII, AMPK, Sirt1

## Abstract

**Background:**

In the early stage of diabetes, the cardiac ejection fraction is preserved, despite the existence of the subclinical cardiac dysfunction to some extent. However, the detailed phenotype of this dysfunction and the underlying mechanism remain unclear. To improve our understanding of this issue, we used low-dose STZ and high-fat diet to induce type 2 diabetic models in rats. The effects and the mechanism associated with the early stages of the disease were analyzed.

**Methods:**

The type 2 diabetic mellitus (T2DM) in SD rats were induced through 30 mg/kg STZ and high-fat diet. Two-dimensional spackle-tracking echocardiography (STE) and the dobutamine test were performed to examine the cardiac function. Calcium transients of left ventricular myocytes were detected and the related intracellular signalling factors were analyzed by western blotting.

**Results:**

After 6-weeks, T2DM rats in left ventricular (LV) diastole showed decreased global and segment strain(S) levels (*P* < 0.05), both in the radial and circumferential directions. Strain rate (Sr) abatement occurred in three segments in the radial and circumferential directions (*P* < 0.05), and the radial global Sr also decreased (*P* < 0.05). In the systolic LV, radial Sr was reduced, except the segment of the anterior septum, and the Sr of the lateral wall and post septum decreased in the circumferential direction (*P* < 0.05). Conventional M-mode echocardiography failed to detect significant alterations of cardiac performance between the two groups even after 12 weeks, and the decreased ejection fraction (EF%), fractional shortening (FS%) and end-systolic diameters (ESD) could be detected only under stress conditions induced by dobutamine (*P* < 0.05). In terms of calcium transients in cardiac myocytes, the T_peak_ in model rats at 6 weeks was not affected, while the T_decay1/2_ was higher than that of the controls (*P* < 0.05), and both showed a dose-dependent delay after isoproterenol treatment (*P* < 0.05). Western blot analysis showed that in 6-week T2DM rats, myocardial p-PLB expression was elevated, whereas p-CaMKII, p-AMPK and Sirt1 were significantly down-regulated (*P* < 0.05).

**Conclusion:**

A rat model of T2DM was established by low dose STZ and a high-fat diet. LV deformation was observed in the early stages of T2DM in association with the delay of Ca^2+^ transients in cardiomyocytes due to the decreased phosphorylation of CaMKII. Myocardial metabolism remodeling might contribute to the early LV function and calcium transportation abnormalities.

## Background

Diabetic cardiomyopathy (DCM) can impair cardiac performance, which will eventually develop into global cardiac dysfunction and contribute heavily to the high mortality of diabetes population [1]. Although cardiac dysfunction is one of the main complications in such disease, clinical studies have shown that asymptomatic diastolic and/or systolic dysfunction occurred in the early stage of diabetes with preserved left ventricular (LV) ejection fraction [2, 3]. Therefore, for therapeutic purpose, it is critical to identify the subclinical cardiac dysfunction as early as possible and elucidate the underlying mechanism(s).

Two-dimensional speckle tracking echocardiography (STE) may calculate strain and strain rate to evaluate regional and/or global cardiac wall motion tracks during cardiac cycle [4, 5]. Because of its angle-independency, two-dimensional STE can minimize the variability during the quantification of regional and global ventricular function and is very sensitive for the detection of cardiac dysfunction in the early stages of diabetes [6]. This technology is currently applied to patients with diabetes to monitor cardiac performance; however, although there have had some studies in clinic, the reports about the data on the early stages of this disease in animal models are only a few [7, 8]. To clarify this issue, in the present study, we designed a protocol based on the use of STE to monitor cardiac performance in a type 2 diabetes mellitus (T2DM) rat model, and analyzed the cardiomyocyte intracellular calcium signaling pathway to improve our understanding of the mechanisms underlying the changes that occur during disease development.

## Methods

### Chemicals and animal foods

Joklik’s modified Minimum Essential Medium Eagle (MEM), collagenase (type I), fetal bovine serum (FBS) and STZ were purchased from Sigma. Fluro-4/AM was obtained from Invitrogen, whereas rat insulin ELISA test kit was obtained from Millipore. Standard laboratory animal food was purchased from Guangdong Provincial Medical Experimental Animal Center containing 3 % fat, 20%protein, 55 % carbohydrate with 310 kJ/kg total calorific value. High-fat food was made from the same place, containing15% butter, 10 % egg yolk powder, 2 % cholesterol and 73 % standard food providing 412 kJ/kg calorie for animals.

### Experimental animals

Thirty-six male Sprague–Dawley (SD) rats in age of about 8 weeks with the weight of about 200 g were purchased from Guangdong Provincial Medical Experimental Animal Center. Rats were housed under a 12-h light–dark cycle and were fed with standard chow for 7 days prior to treatment and tap water *ad libitum*. The room temperature was kept at 22 ± 2 °C. All stressful conditions were avoided.

The maintenance of animals was under the Guide for the Care and Use of Laboratory Animals published by the US National Institute of Health (1996). Experimental protocols were approved by the research ethic committee of Guangdong General Hospital.

### Induction and identification of type 2 diabetes

The design of the protocol was came from previous reports [9, 10] and was amended slightly. In brief, after one week of normal feeding, rats were randomly divided into two groups: control (16 rats) and model (20 rats). Twelve hours before treatment, food was withdrawn from housing cages except for the water supply. In the model group, rats were injected with 30 mg/kg of STZ from tail vein, which was prepared in 0.1 M citrate buffer (pH4.5) right before use. Then, the animals were fed with the high-fat diet. In the control group, rats were injected with the same solution of the model but with the absence of STZ and fed with normal diet as before.

Fasting plasma glucose (FPG), random plasma glucose (RPG) measurement and oral glucose tolerance test (OGTT) were performed to monitor the change of the concentration of blood glucose in rats. Initially, before fasted, blood sample from tail vein of rats after 7 days of induction treatment was obtained to detect the RPG, and then all rats were starved for further experiments. After 12-h overnight starvation, blood samples for test were collected from rat’s tail vein at 0, 30, 60, 90,120mins after glucose lavage (at 1 g/kg of body weight). Fasting serum samples were also collected for insulin concentration analysis by ELISA assay and plasma glucose concentrations were measured through Roche Accu-Chek Performa.

### Traditional echocardiography

At 6 weeks, rats were anaesthetized with isoflurane (1.5 % isoflurane and 98.5 % O_2_) and placed on a heating pad in supine position. Echocardiography images were obtained with 16 MHz detector (VevoStrain 2100, VisualSonics, Toronto, ON, Canada). In each group, left ventricle M-mode images were acquired from parasternal short axis view at the mid papillary muscle level. Measurement included end-diastolic and end-systolic LV anterior wall thickness (AWD and AWS, respectively), end-diastolic posterior wall thickness (PWD) and diameters (EDD), end-diastolic and end-systolic LV volumes (EDV and ESV, respectively), ejection fraction (EF) and fractional shortening (FS). In addition, diastolic function was assessed using colourful Doppler across the mitral valve from the apical four chamber views. In this case, the E/A ratio from early (E) and late (A) flow velocity and mitral valve deceleration time (MVDT) indicated diastolic function.

### Two-dimensional speckle tracking echocardiography

After being acquired M-mode still images, rats were then taken the strain and strain rate measurements through two-dimensional STE images (100 frames per second) acquired from parasternal short-axis view on mid papillary level in B-mode as previously described [11]. Strain and strain rate were both quantified in circumferential and radial axes. In brief, two-dimensional image was frozen at the end of systole and endocardial border was carefully and manually traced at LV parasternal short-axis views, and then epicardium was covered automatically. Tracked images were divided into six segments including anterior free wall (AFW), lateral wall (LW), posterior wall (PW), inferior free wall (IFW), posterior septum (PS) and anterior septum (AS). Then, the peak endocardial systolic radial and circumferential strain and strain rate of each segment were analyzed and calculated by Vevo Strain software. Global strain and strain rate were the average of all six segments. Finally, diastolic and systolic LV function was evaluated by the average of three independent cardiac cycles.

### Dobutamine stress test

In 12 weeks, rats were given dobutamine to evaluate the cardiac function under stress condition using M-mode echocardiography. Based on Planet’s protocol, cannulation and infusion of 10 g/kg/min of dobutamine from the tail vein was applied in this test [12]. M-mode still images of all animals in test were acquired initially and then time lapse images were captured every two minutes during the entire infusion period. Finally, images of the whole process were analyzed and compared.

### Isolation of left ventricular cardiac myocytes

Cardiac myocytes from rat left ventricular at 6 weeks were isolated by retrograde enzymatic perfusion as previously described with slight modification [13]. In brief, the rat hearts were quickly excised and fixed on the Langendorff apparatus. In the initially 7 ~ 8 min, isolated hearts were perfused with Ca^2+^-free Joklik medium working solution (with HEPES added and dissolved in diluted water followed the instruction, and adjusted to pH7.2) at 2–3 ml/min to wash out the remnant blood. The perfusion was then followed the same solution containing 0.5 mg/ml of collagenase and 0.1 % of BSA for further 35 min. After perfusion the ventricles were sheared, gently torn up into fragments and the cells were washed in Joklik’s modified MEM. Subsequently, the suspension was washed three times and a CaCl_2_ solution was gradually added to the final concentration of 1.25 mM by 8 steps with 5-min interval. Finally, the myocyte suspensions were stored at room temperature and the following experiments were performed within 6 h.

### Measurement of calcium transient

Isolated cardiac myocytes were loaded onto a calcium indicator, 1.0 μmol/L Fluo-4/AM at room temperature, lucifugal. Twenty minutes later, cells were washed twice and resuspended in physiological buffer containing 132 mM NaCl, 4.8 mM KCl, 1.2 mM MgCl_2_, 5 mM glucose, 10 mM HEPES and 1.25 mM CaCl_2_. Then, the suspended cells were placed in a superfusion chamber on the laser scanning confocal microscope system (LSCM, Leica, SP5-FCS, Germany). Through a pair of platinum electrodes positioned in the chamber, myocytes were initially field-stimulated at 1 Hz at least for 1 min before measured for minimizing the differences, and then 60 Hz (1 min pulse duration, 5–10 % above threshold) was applied as the basic stimulating condition. One minute later, isoproterenol was added with the final concentration of 10^−9^, 10^−8^, 10^−7^, 10^−6^ M stepwise. During each stimuli, the movement and fluorescent of interest area was monitored continuously by LSCM. The optical area was restricted of only a single cell during one experiment duration with a line through the largest diameter and recorded under the x-t manner. Fluo-4 fluorescent was measured at 525 nm and its time curve was recorded automatically.

### SDS-polyacrylamide gel electrophoresis (PAGE) and immunoblotting

Left ventricular myocardium of rats in 6 weeks was lysed in RIPA buffer containing phosphotase and protease inhibitors. Protein concentrations were quantified using Bio-Rad (Biorad Laboratories, Hercules, CA, USA). Equal amounts of protein were loaded on 8–15 % polyacrylamide gels and subjected to SDS-PAGE, then electrotransferred onto PVDF membranes (Millipore Corporation, Bedford, MA, USA) and immunoblotted using: anti-phosphoryl-phosphlamban (Ser16) (rabbit polyclonal, Millipore, #07052), anti-phosphoryl-phosphlamban (Thr17) (rabbit polyclonal, Santa Crutz, sc-17024-R), anti-phospholamban (rabbit polyclonal, Cell Signaling,#8495); anti-phosphoryl-CaMKII (T287) and anti-CaMKII (rabbit polyclonal, abcam, ab32678 and ab52476); anti- phosphoryl-AMPKα(Thr172) and anti-AMPKα (rabbit polyclonal, Cell Signaling, #2513 and #2532); anti-ATP2a/SERCA2 (rabbit mAb, Cell Signaling, #9580), anti-Sirt1 (mouse monoclonal, Cell Signaling, #8469) antibodies. All antibodies were used in 1:1000 dilutions unless indicated otherwise in the text. Antigen-antibody complexes were detected using anti-rabbit or anti-mouse antibodies conjugated to horseradish peroxidase (HRP) and visualized using SuperSignal chemiluminescent detection kit (Pierce, Rockford, Ill, USA). HRP-conjugated anti-GAPDH (Kangcheng, China,KC-5G5) and α-tubulin (Cell Signaling, #2144) was used to ensure equal loading of protein samples.

### Masson’s TRICHROME STAINING analysis

Left ventricular wall of 6 and 12 weeks rats were fixed with 10 % formalin and embedded in paraffin. Slices were serially cut within 5 μm thickness and were all cross sections. Then the instruction of Masson’s Trichrome stain kit was followed and microscope was used to get the pictures.

### Statistic analysis

All values were presented as the mean ± standard deviation (SD). Data were subjected to Student’s *t* test for two-way unpaired analysis of variance between two groups using GraphPad Prism 1.1 program. The *P* values less than 0.05 were considered significant.

## Results

### Induction of type 2 diabetes in rats through the low-dose of STZ and high-fat diet

**Fig. 1:**
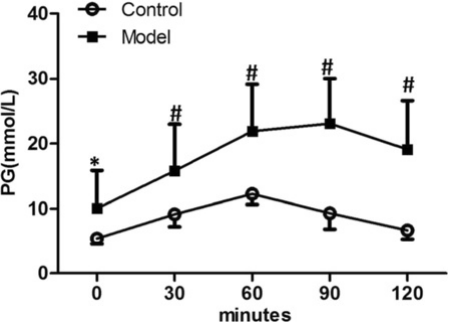
The time course curve of OGTT of rats in 7 days after the induction treatment. Data are presented as mean ± SD. Plasma glucose of model rats was significantly higher than that of the control at all time points before and after glucose lavage. * *P* < 0.05; # *P* < 0.01

Seven days after injection of STZ, blood samples were collected from tail vein. The successful induction of diabetes was confirmed by measuring the fasting blood glucose concentration (FPG) and random blood glucose concentration (RPG) in model rats. According to the diagnosis standard of type 2 diabetes, rats with RPG level over 11 mM and/or FPG level over 7 mM in two consecutive measurements were considered as diabetic. On day 7, 16 out of 20 rats started to show signs of diabetes. Therefore, the induction successful rate was 80 %. By the end of 3 weeks, the average levels of fasting plasma glucose (FPG) and 2-h plasma glucose (2 h PG) in control group were 5.4 ± 1.0 mmol/L and 6.9 ± 1.2 mmol/L, respectively, whereas the same values were increased 2–3 folds in the model group. OGTT and ELISA test showed that at all time points, the plasma glucose levels in model rats were higher than that of the control (*P* < 0.05) (Fig. 1) but insulin level in serum did not differ significantly (*P* > 0.05), indicating that these animals had acquired T2DM (Table 1). Then, the diabetic condition was stably maintained and closely monitored. All DM rats appeared to be otherwise healthy and survived for at least 6 weeks. One T2DM rat died at about 10 weeks after disease induction, and another one died approximately 13 min after dobutamine infusion in a later test. None of the rats in control group died during the study period.

**Fig. 2:**
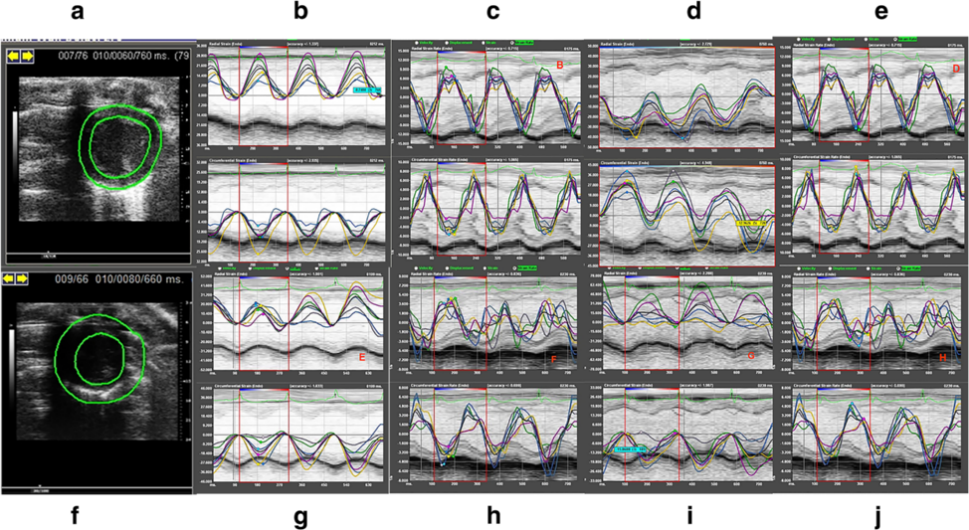
**a**-**e**: typical analysis pictures of radial and circumferential strain and strain rate from the view of parasternal short axis (at the mid papillary muscle level) of control rats; **f**-**j**: typical analysis pictures of radial and circumferential strain and strain rate from the view of parasternal short axis (at the mid papillary muscle level) of model rats

**Table 1:** Blood glucose level of rats at 3 weeks. Data are presented as mean ± SD.

Group	Size	Fasting plasma glucose	Two-hrs plasma glucose	Serum insulin
		(mmol/L)	(mmol/L)	(ng/ml)
Control	16	5.4 ± 1.0	6.9 ± 1.2	3.32 ± 1.01
Model	16	10.1 ± 7.3^#^	18.8 ± 7.6^#^	2.83 ± 1.07

^#^Compared to control group, *P*<0.01

### Detection of early diabetic cardiac dysfunction by echocardiography

**Fig. 3:**
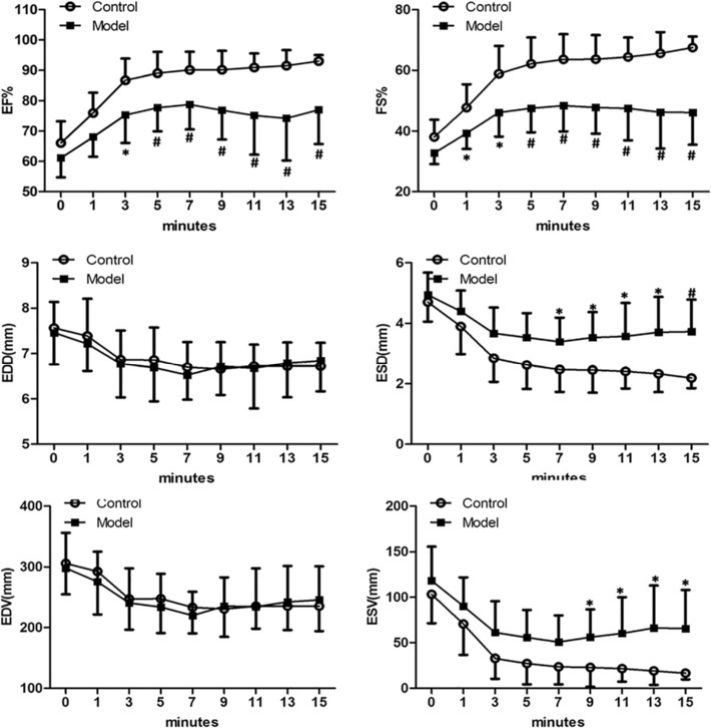
M-mode echocardiography measurement of left ventricular of rats during dobutamine infusion (10 g/kg/min) from tail intravenous at 12 weeks. The statistically differences of EF%, FS%, ESD and ESV between the two groups could be detected. * *P* < 0.05; # *P* < 0.01; Abbreviations: EF = ejection fraction; FS = fractional shortening; EDD = end-diastolic diameter; ESD = end-systolic diameter; EDV = end-diastolic volume; ESV = end-systolic volume

To compare different methods for detection of diabetic cardiac performance, half of the control and model rats were examined firstly by traditional M-mode and colour Doppler to evaluate the LV systolic and diastolic function (Fig. 2). As shown in Table 2, the EF% and FS% were slightly reduced about 6 and 8 %, respectively, in model group at 6 weeks, and 11 and 14 %, respectively, at 12 weeks. However, the differences were not statistically significant. The average thickness of LV anterior wall was nearly identical between control and model groups at 6 and 12 weeks. Also, there was no significant difference between the control and model groups in either end-diastolic and end-systolic diameters or volumes. Meanwhile, the E/A ratio did not differ significantly between the two groups. Furthermore, when the model rats were treated with dobutamine infusion, the levels of EF% and FS% were steadily increased in control group but to a much less extent than that in the model group (Fig. 3). The diastolic function as measured by LV diameter and volume, was relatively stable, whereas systolic function was increased in the control group. The model rats showed comparable diastolic function to that of the control group, whereas they showed a lower capacity for systolic reaction under stress conditions, which indicated cardiac dysfunction.

**Table 2:** Measurements of left ventricular (LV) function in rats by M-mode echocardiography from the parasternal short axis at the mid papillary muscle level, and colourful-Doppler across the mitral valve from the apical four chamber views

	6 Weeks		12 Weeks		*P*-value			
	Control Rats	Model Rats	Control Rats	Model Rats	*P1*	*P2*	*P3*	*P4*
	(*n* = 8)	(*n* = 8)	(*n* = 8)	(*n* = 7)				
AWD(mm)	1.82 ± 0.20	1.82 ± 0.17	1.74 ± 0.22	1.74 ± 0.25	0.9445	0.4912	0.7806	0.3278
AWS (mm)	2.86 ± 0.39	2.89 ± 0.32	2.59 ± 0.38	2.43 ± 0.19	0.9521	0.1701	0.2972	0.0091
PWD (mm)	2.15 ± 0.22	2.01 ± 0.33	1.87 ± 0.19	1.70 ± 0.23	0.3284	0.1142	0.0230^*^	0.0652
EDD (mm)	7.44 ± 0.39	7.69 ± 0.99	7.47 ± 0.57	7.58 ± 0.57	0.5227	0.7959	0.6183	0.6498
EF (%)	72.61 ± 6.38	68.19 ± 8.64	66.37 ± 7.06	59.34 ± 5.27	0.2641	0.0629	0.0724	0.0651
FS (%)	43.33 ± 5.46	39.82 ± 7.03	38.22 ± 5.74	32.69 ± 3.65	0.2842	0.0613	0.0748	0.0330^*^
EDV (ml)	293.78 ± 32.90	321.15 ± 93.93	308.12 ± 51.21	299.13 ± 58.17	0.4496	0.8122	0.5800	0.6012
ESV (ml)	80.28 ± 19.33	106.38 ± 48.40	105.11 ± 31.42	124.43 ± 35.19	0.1784	0.2979	0.0782	0.4485
E/A Ratio	1.54 ± 0.17	1.83 ± 0.51	1.68 ± 0.26	1.56 ± 0.18	0.1477	0.3586	0.2440	0.2109
MVDT(ms)	14.17 ± 5.80	17.45 ± 8.91	16.03 ± 2.56	16.77 ± 2.96	0.3969	0.6258	0.4473	0.8504

Data are presented as mean ± SD. *Abbreviations AWD* anterior wall in diastole, *AWS* anterior wall in systole, *PWD* posterior wall in diastole, *EDD* end-diastolic diameter, *EF* ejection fraction, *FS* fractional shortening, *EDV* end-diastolic volume, *ESV* end-systolic volume, E/A Ratio ratio of the early (E) to late (A) ventricular filling velocities, *MVDT* mitral valve deceleration time

*P*1 and *P*2, Model group compared to control group; *P*3 and *P*4, 12-week compared to 6-week group, *P<0.05

In parallel, the remaining control and model rats were subjected to two-dimensional STE analysis. Strain and strain rate in both control and model groups at 6 weeks are shown in Table 3. During the diastolic phase, S and Sr in radial and circumferential direction were all affected in the model rats. Control and model rats showed significant differences (*P* < 0.05) in global and all segmental strain, as well as partially in some segmental strain rate During the systolic phase, differences between the control and model rats were observed in both radial and circumferential strains and strain rates, although most of the differences (except LW and PW strain rates) were not statistically significant. These results indicated the presence of diastolic dysfunction in model rats in the early days of the disease, which can be detected by the two-dimensional STE.

**Table 3:** Peak regional and global strain and strain rate of rats at 6 weeks measured through STE. Views were detected from the parasternal short axis at the mid papillary muscle level

			Strain			Strain Rate		
			Control Rats	Model Rats	*P*	Control Rats	Model Rats	*P*
			(*n* = 8)	(*n* = 8)		(*n* = 8)	(*n* = 8)	
Diastolic	Radial	AFW	−36.04 ± 12.68	−9.05 ± 10.10	<0.001^#^	−11.06 ± 4.14	−7.63 ± 1.99	0.1144
		LW	−24.86 ± 4.25	−10.16 ± 7.58	<0.001^#^	−9.62 ± 2.87	−5.79 ± 1.37	0.0087^#^
		PW	−25.03 ± 11.78	−10.39 ± 9.79	0.0076^#^	−8.37 ± 3.04	−5.58 ± 1.77	0.0299^*^
		IFW	−29.12 ± 13.97	−11.21 ± 12.85	0.0126^*^	−8.52 ± 2.03	−6.64 ± 1.78	0.0453^*^
		PS	−22.88 ± 12.69	−6.24 ± 4.63	0.0031^#^	7.07 ± 2.99	−5.78 ± 2.03	0.2376
		AS	−21.64 ± 13.99	−4.24 ± 4.75	0.0209^*^	−6.89 ± 2.27	−7.07 ± 1.71	0.7496
		Average	−26.63 ± 9.04	−7.05 ± 5.97	<0.001^#^	−9.13 ± 2.51	−6.62 ± 1.16	0.0304^*^
	Circumferential	AFW	26.29 ± 12.24	1.43 ± 0.99	<0.001^#^	7.64 ± 2.77	4.44 ± 2.45	0.0417^*^
		LW	27.35 ± 13.97	2.95 ± 2.63	<0.001^#^	7.31 ± 2.73	3.14 ± 2.02	0.0045^#^
		PW	31.07 ± 11.35	0.80 ± 0.34	<0.001^#^	8.06 ± 1.71	5.36 ± 1.92	0.0094^#^
		IFW	29.44 ± 19.96	0.81 ± 1.04	<0.001^#^	10.53 ± 6.33	5.74 ± 2.93	0.0789
		PS	29.77 ± 19.52	0.90 ± 0.46	0.0010^#^	8.10 ± 2.44	8.75 ± 6.02	0.7597
		AS	21.32 ± 8.25	0.66 ± 0.40	<0.001^#^	6.25 ± 2.31	5.96 ± 1.80	0.9022
		Average	29.57 ± 14.35	1.25 ± 0.69	<0.001^#^	7.98 ± 2.37	5.65 ± 2.55	0.1008
Systolic	Radial	AFW	38.72 ± 10.04	23.92 ± 9.61	0.0537	9.79 ± 6.05	4.76 ± 1.79	0.0407^*^
		LW	26.89 ± 14.94	19.44 ± 8.94	0.2461	7.76 ± 3.56	4.04 ± 1.83	0.0229^*^
		PW	25.87 ± 14.55	19.77 ± 10.01	0.3456	7.15 ± 1.54	3.97 ± 1.75	0.0012^#^
		IFW	24.83 ± 10.11	17.33 ± 10.48	0.1671	8.20 ± 3.16	4.30 ± 2.44	0.0158^*^
		PS	16.91 ± 6.25	10.63 ± 8.49	0.1144	6.93 ± 1.84	4.13 ± 2.82	0.0211^*^
		AS	25.73 ± 19.00	19.19 ± 7.47	0.3801	5.71 ± 2.15	4.02 ± 2.33	0.1817
		Average	26.13 ± 9.75	18.12 ± 5.56	0.0630	8.67 ± 2.59	4.23 ± 1.94	0.0022^#^
	Circumferential	AFW	−23.39 ± 8.39	−15.77 ± 9.22	0.1495	−6.74 ± 2.81	−4.86 ± 3.78	0.3395
		LW	−20.64 ± 6.31	−10.34 ± 12.62	0.0584	−6.54 ± 2.31	−2.37 ± 1.29	0.0010^#^
		PW	−21.98 ± 4.79	−19.57 ± 8.18	0.5102	−7.11 ± 1.41	−5.26 ± 1.24	0.0238^*^
		IFW	−24.04 ± 7.22	−21.58 ± 10.30	0.3488	−7.42 ± 2.52	−5.45 ± 1.62	0.1154
		PS	−23.00 ± 3.23	−27.95 ± 10.02	0.2074	−8.25 ± 2.92	−7.68 ± 4.87	0.8873
		AS	−19.84 ± 7.12	−24.72 ± 8.53	0.1340	−5.61 ± 1.78	−6.60 ± 3.31	0.3478
		Average	−22.01 ± 3.87	−19.99 ± 7.67	0.5314	−6.95 ± 1.98	−5.65 ± 3.18	0.4198

Data are presented as mean ± SD. *Abbreviations*: *AFW* anterior free wall, *LW* lateral wall, *PW* posterior wall, *IFW* inferior free wall, *PS* post septum, *AS* anterior septum

*Compared to control group, *P*<0.05; #Compared to control group, *P*<0.01

### Impaired Ca^2+^ handling in diabetic rats and its possible molecular mechanisms

**Fig. 4:**
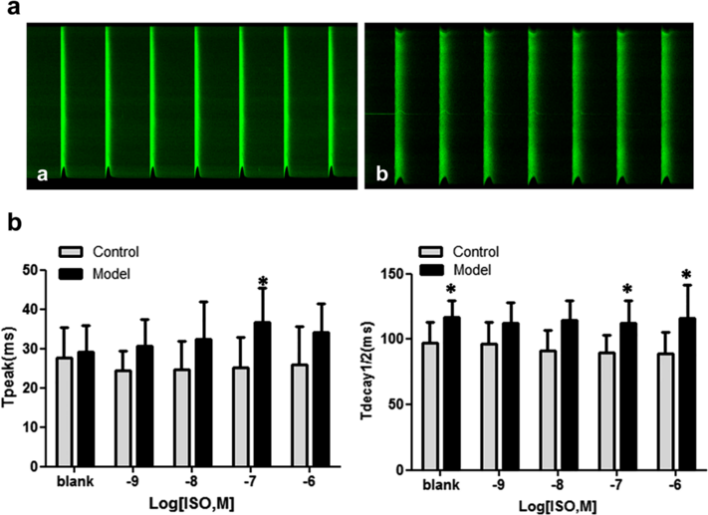
The calcium transient images before ISO treatment and values of isolated rat cardiomyocytes at 6 weeks. In control group, 32 cells were measured from 5 rats, whereas in model group 36 cells were measured from the same number of rats. Cells were electrically paced with 60 Hz current with or without isoprenaline (ISO) treatment. **a**: calcium transient image of cardiomyocyte of the control rat; **b**: calcium transient image of cardiomyocyte of the model rat. Data are presented as mean ± SD. Abbreviations: T_peak_ = Time to peak; T_decay1/2_ = Time of 50 % decay from peak. Compared the control, the T_decay1/2_ of cardiomyocytes of model rats was significantly increased before and after ISO treatment. * *P* < 0.05; # *P* < 0.01

The Ca^2+^ handling were examined in diabetic rats. To this end, cardiomyocytes were isolated from both control and model rats at 6 weeks and the Ca^2+^ transient was measured as described in Materials and Methods. As shown in Fig. 4, the time to peak (T_peak_) of the Ca^2+^ transient was similar between control and model rats, but the time of 50 % decay from peak (T_decay1/2_) was significantly delayed in model rats (*P* < 0.05). To investigate the calcium handling capacity of cells under stress, cells were then treated with different dose of isoproterenol (ISO). The results showed that at higher ISO dosages, there were significant differences between cells from control and model groups (*P* < 0.05). These results suggested that Ca^2+^ handling was impaired in cardiomyocytes of model rats.

**Fig. 5:**
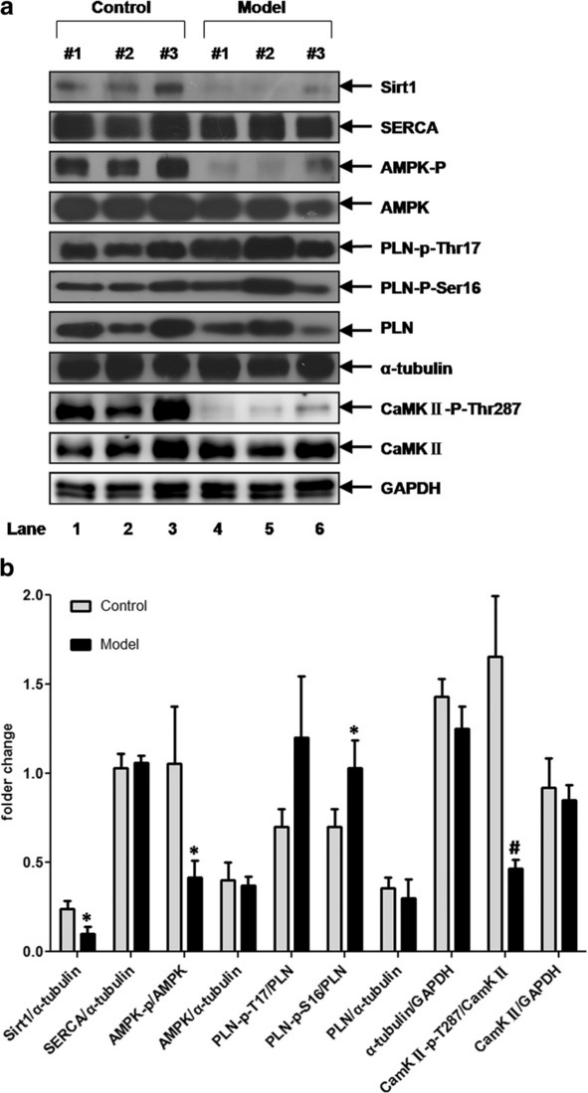
Western blotting analysis of the expression of key players of calcium transient and AMPK, Sirt1 in LV myocardium of rats. Data are presented as mean ± SD. GAPDH and α-tubulin were performed to ensure the equal loading of protein samples. Compared the control, the expression of PLN-p-S16 in model rats’ LV myocardium was increased whereas the Sirt1, AMPK-p and CaMKII-p-Thr287 were all decreased remarkably. * *P* < 0.05; # *P* < 0.01

The cellular proteins involved in the regulation of calcium transients and diabetic cardiac dysfunction in these animals were analyzed by western blotting. In this regard, As shown in Fig. 5, the expression of SERCA did not differ between control and model rats, whereas the phosphorylation of PLB at Ser 16 elevated significantly and somewhat increased at Thr17. On the other hand, the phosphorylation of CaMKII was decreased more than 70 % (*P* < 0.05). The detection of the expression of adenosine 5’-monophosphate (AMP)-activated protein kinase (AMPK) and its key regulator Sirt1 showed that the total protein level of AMPK remained unchanged in model rats, whereas the phosphorylation of AMPK at Thr172 and the expression of Sirt1were both reduced by approximately 50 % (*P* < 0.05) (Fig. 4).

## Discussion

In many aspects, the cardiac dysfunction characteristic of type 1 and type 2 diabetes in humans can be reproduced in rodent models [8]. Most studies to date have focused on the use of transgenic mice. Consistent with previous studies [9, 10], our data showed that low-dose (30 mg/kg) STZ and a high-fat diet could induce T2DM in rats, and the blood insulin test results (Table 1) showed that this low dose of STZ did not significantly affect the insulin secretion function of islet beta-cells. Therefore, the ability of this model to mimic T2DM in rats makes it suitable for research studies. In the present study, the intraperitoneal injection of STZ was also replaced by tail vein. This replacement ensured the complete drug absorption, led to an 80 % success rate of induction of the disease only after 7 days of the treatment, which was much more efficient than what was in showed in the previous report.

In this study, M-mode echocardiography detected somewhat weak signs of systolic dysfunction in the early stages of disease (Table 2). Compared rats in 6-weeks, only the PWD of the control and FS% of the model in 12-weeks decreased, it showed that the effect of prolongation of the age or hyperglycaemic status on the cardiac function were still not obvious. And the differences between the two groups, for certain parameters, such as EF% and FS%, the levels in model rats were slightly lower than those of the controls. The change was not significant until 12 weeks, and the difference was detected only after dobutamine inducing. No significant differences in the E/A ratio were detected between the two groups. By contrast, the LV deformation in model rats was detected by STE at 6 weeks. These results demonstrated that the sensitivity of STE versus conventional M-mode measurements. As a simpler and more accessible measurement method, M-mode echocardiography remains the first option for cardiac function evaluation in the clinic; however, the significance of STE has been demonstrated and the method has been gradually utilized, especially for data collection on the cardiac function of patients with preserved ejection fraction [7]. Evidence obtained through the use of this technology in the clinic has demonstrated the existence of subclinical cardiac dysfunction in the early stages of T2DM. However, there are few studies using STE in small animals, including the present work. Previously, in Li’s report [11], LV deformation in systole could be detected in db/db mice at 16 weeks of age. Our data showed that in T2DM diabetic rats induced by low-dose STZ and a high-fat diet, although the LV strain in systole at 6 weeks remained unchanged, the strain rate in some segments and the global strain rate in the radial direction could both be detected (Table 3). This indicated the different characteristics of the disease development in this model compared with that in db/db mice. Furthermore, our analysis showed that in this period of the disease, not only the deformation of the LV could be found in systole, but also in diastole, and the latter was more severe than the former. These findings are consistent with clinical trends, which show that in T2DM patients, the cardiac diastolic dysfunction occurs earlier than the systolic dysfunction.

**Fig. 6:**
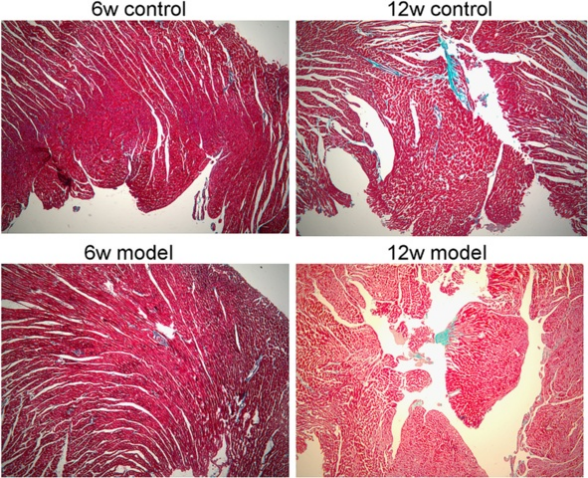
Masson’s Trichrome Staining analysis. The normal cardiomyocytes were stained red, and fibrotic areas were stained green as usual. The results showed that till 12 weeks there was no significant fibrosis could be observed in model rats myocardium. Pictures were taken with 5 × objective lens

The contractility-relaxation of cardiomyocytes and the condition of myocardial connective tissues affect the motion of the cardiac wall. Generally, connective tissue deposition is a late event during the development of DM. In the present study, histological analysis revealed few, if any, sign of fibrosis in diabetic rats in the early weeks (Fig. 6). Meanwhile, we found that the Ca^2+^transients of left ventricular cardiomyocytes were delayed in these animals (Fig. 4); this was significant in theT_decay1/2_ and even more evident upon ISO stress. These findings suggested that in the early stage of our model rats, the LV deformation may be due to the affected cardiomyocyte relaxation-contractility caused by the intracellular calcium handling damage. SERCA-2a acts as a key factor in the uptake of Ca^2+^into the sarcoplasmic reticulum (SR) which determines the intracellular calcium transportation dynamics and thus the cardiomyocyte relaxation-contractility. Previous studies have reported that in diabetic animals, the protein or activity level of SERCA-2a was decreased contributing to the cardiac dysfunction, but these reports was mainly focused on type 1 models and/or type2 diabetic rats at later stages of the disease [14–17]. In the present study, in T2DM rats at 6 weeks, the expression of SERCA-2a in cardiomyocytes remained unchanged, and its key modulator, PLB, showed a higher phosphorylation rate (Fig. 4). This indicated the possible up-regulation of the activity of the SERCA pump and an improved calcium transportation capability. Similar findings has been reported by Miklo et al [18], showing that in 6-week metabolic syndrome rats, LV cardiomyocytes were declined in Ca^2+^ sequestration capacity, while SERCA activity remained intact. These facts imply that in the early stage of the fat-overloaded and hyperglycemic status, the uptake of Ca^2+^ to the SR may be delayed, which affects the normal processes of cardiomyocyte relaxation-contractility; whereas SERCA, and its primary regulator PLN, may remain normal or even stronger to compensate for this abnormality. On the other hand, an interestingly thing is that in the present study, additional experiments found that in these T2DM rats, CaMKIIphosphorylation was attenuated remarkably in the LV myocardium. CaMKII is an important molecule that plays a role in calcium homeostasis. It can modulate PLN and RyR function through phosphorylation, and studies found that this protein also has an autophosphorylating function at Thr287 to confer Ca2+/CaM-independent activity. When the autophosphorylated isoform is activated, it can sustain CaMKII activity even in the absence of elevated Ca2+/CaM, thus extending the CaM-dependent regulation [19]. However, whether CaMKIIabnormality affects cardiac function in T2DM remains unclear. Our findings indicate that in the early stages of T2DM, the activity of CaMKIIis decreased, which could be attributed to alterations in calcium homeostasis in the heart caused by its down-regulation and the consequent inhibition of downstream targets such as PLN. Due to the limitations of our experiments, at present we were unable to provide more evidence supporting our thoughts, but in previous report [20], study found that the depression of Thr287-autophosphorylation could lead to calcium transient delay and cardiomyocyte diastolic function, contributing to the increased arrhythmia, hence this fact might be a circumstantial evidence to support this hypothesis.

In T2DM, the myocardium energetic metabolism was remodelled, which could affect the capability of calcium homeostasis of cardiomyocytes [21, 22]. AMPK is a major regulator of cardiac metabolism, including lipid oxidation and glucose uptake [23], and the suppression of AMPK signalling pathway is found in the process of diabetic cardiomyopathy. In addition, AMPK can activate cardiac troponin I (cTnI), which has a direct positive effect on myocardial contractility at the myofilament level [24]. In this study, we found that the expression level of AMPK was not affected, but its phosphorylation status was remarkably diminished during the onset of T2DM in rat, similar to the expression of its positive regulator Sirt1 (Fig. 4). These findings suggested that metabolic remodelling was involved in the initial stage of the process of T2DM in rats. Reports have shown an inverse relationship between the cardiac diastolic strain rate and the myocardial triglyceride content through strain-encoded magnetic resonance analysis of T2DM patients, although the patients’ systolic function was preserved [25, 26]. This clinical information taken together with our results indicates that there is a relationship between energy remodeling and myocardial deformation, and the cardiomyocyte intracellular SIRT1/AMPK signaling pathway may play a role in this association.

## Conclusions

In conclusion, in the present study, a rat model of T2DM was established by low dose STZ and a high-fat diet. Using these model rats, LV deformation occurred at the early stages of the disease and was associated with calcium transient delay. The downregulation of the activity of the CaMKII and SIRT1/AMPK signaling pathways could be attributed to cardiomyocyte intracellular calcium handling impairment and myocardial deformation. These results suggest a role for metabolic remodeling in the onset of DCM in rats, and the current animal model of T2DM provides a useful tool for further investigation of the etiology of this disease.

## Abbreviations

STZ: streptozotocin
DM: diabetic mellitus
STE: spackle-tracking echocardiography
EF: ejection fraction
FS: fractional shortening
LV: left ventricular
AWD: anterior wall in diastole
AWS: anterior wall in systole
PWD: posterior wall in diastole
EDD: end-diastolic diameter
EDV: end-diastolic volume
ESV: end-systolic volume
E/A Ratio: ratio of the early (E) to late (A) ventricular filling velocities
MVDT: mitral valve deceleration time
S: strain
Sr: strain rate
AFW: anterior free wall
LW: lateral wall
PW: posterior wall
IFW: inferior free wall
PS: post septum
AS: anterior septum.
